# Unsteady hybrid-nanofluid flow comprising ferrousoxide and CNTs through porous horizontal channel with dilating/squeezing walls

**DOI:** 10.1038/s41598-021-91188-1

**Published:** 2021-06-16

**Authors:** Muhammad Bilal, Hamna Arshad, Muhammad Ramzan, Zahir Shah, Poom Kumam

**Affiliations:** 1 Department of Mathematics, The University of Lahore, Gujrat Campus, Gujrat, Pakistan; 2 Bahria University, Islamabad, Pakistan; 3 Department of Mathematical Sciences, University of Lakki Marwat, Lakki Marwat, 28420 Khyber Pakhtunkhwa Pakistan; 4 Center of Excellence in Theoretical and Computational Science (TaCS-CoE), Faculty of Science, King Mongkut’s University of Technology Thonburi (KMUTT), 126 Pracha Uthit Rd., Bang Mod, Thung Khru, Bangkok, 10140 Thailand; 5 Fixed Point Research Laboratory, Fixed Point Theory and Applications Research Group, Center of Excellence in Theoretical and Computational Science (TaCS-CoE), Faculty of Science, King Mongkut’s University of Technology Thonburi (KMUTT), 126 Pracha Uthit Rd., Bang Mod, Thung Khru, Bangkok, 10140 Thailand; 6 Department of Medical Research, China Medical University Hospital, China Medical University, Taichung, 40402 Taiwan

**Keywords:** Mathematics and computing, Physics

## Abstract

The key objective of the present research is to examine the hybrid magnetohydrodynamics (MHD) nanofluid (Carbon-nanotubes and ferrous oxide–water) *CNT*–*Fe*_3_*O*_4_/*H*_2_ flow into a horizontal parallel channel with thermal radiation through squeezing and dilating porous walls. The parting motion is triggered by the porous walls of the channel. The fluid flow is time-dependent and laminar. The channel is asymmetric and the upper and lower walls are distinct in temperature and are porous. With the combination of nanoparticles of *Fe*_3_*O*_4_ and single and multi-wall carbon nanotubes, the hybrid nanofluid principle is exploited. By using the similarity transformation, the set of partial differential equations (PDEs) of this mathematical model, governed by momentum and energy equations, is reduced to corresponding ordinary differential equations (ODEs). A very simple numerical approach called the Runge–Kutta system of order four along with the shooting technique is used to achieve the solutions for regulating ODEs. MATLAB computing software is used to create temperature and velocity profile graphs for various emerging parameters. At the end of the manuscript, the main conclusions are summarized. Through different graphs, it is observed that hybrid-nanofluid has more prominent thermal enhancement than simple nanofluid. Further, the single-wall nanotubes have dominated impact on temperature than the multi-wall carbon nanotubes. From the calculations, it is also noted that *Fe*_2_*O*_3_–*MWCNT*–*water* has an average of 4.84% more rate of heat transfer than the *Fe*_2_*O*_3_–*SWCNT*–*water*. On the other hand, 8.27% more heat flow observed in *Fe*_2_*O*_3_–*SWCNT*–*water* than the simple nanofluid. Such study is very important in coolant circulation, inter-body fluid transportation, aerospace engineering, and industrial cleaning procedures, etc.

## Introduction

We are living in the age of machines. To improve the mechanism and functioning of the cooling system of these machines, a new technology of insertion of the nano-sized particles in the base fluid was introduced a few years ago. Nanofluids are used to enhance the heat transfer rate and thermal conductivity of the base fluid. Normally base fluids are tri-ethylene–glycol, water, refrigerants, ethylene, lubricants and oils, polymeric solutions, and bio-fluids. The common nanoparticle such as copper, gold, silver, alumina, titania, zirconia, *Al*_2_*O*_3_, *CuO* metal carbides *SiC*, metal nitride *AIN*, *SiN*, the carbon in the forms of graphite, diamond, carbon nanotubes, and some other functionalized nanoparticles are used. Nanofluids have unique properties like homogeneity, high thermal conductivity at lower nanoparticle aggregation, stability for a long period, and very little clogging in flow passages. That's why these fluids have a large number of applications in electrical appliances such as micro-electromechanical systems, cooling of microchips, micro-reactors and fluidic digital display devices, etc. Nanofluids are also useful in heating and cooling of buildings, heat interchangers, transportation industry, sensing, microfluidics, lubricant systems, pharmaceutical processes, nano cryosurgery, refrigeration of electronic apparatus, cancer therapeutics, nano-drug delivery, cryopreservation, and imaging^1–7^.

Ferrofluids are a famous kind of nanofluid formed by the insertion of nanoparticles containing iron like Cobalt ferrite, Hematite, and magnetite having a size range of 5–15 nm coated with a layer of surfactant in the base fluid. Ferrofluids have dual properties, as it acts as liquid and magnetic solids at the same time. If the magnetic field is applied across the ferrofluids, we can control the flow of fluid and the rate of heat transfer. Ferrofluids have vast applications in the industrial area such as vacuum chambers in the semiconductor industry, in gravity gradient satellites as viscous dampers, rotating X-ray anode generators, accelerometer, and energy conversion devices, to remove the dust particles in high-speed computers and to eliminate the other impurities from biomedical industries. Zaheer and Mariam^8^ presented the stagnation point flow of ferrofluids having heterogeneous and homogeneous reactions along with non-linear slip conditions. They reported that the velocity component is higher for ferrofluid as compared to the pure base fluid. Abid et al.^9^ scrutinized the inertial and microstructure properties of ferrofluids in the presence of thermal conductivity. They also observed that the velocity of ferrofluids is much greater than the simple base fluid. The MHD flow of ferrofluids with heat flux along with the stretching cylinder was studied by Qasim et al.^10^. During their work, they found that in the presence of a magnetic field, the heat transfer rate of magnetic nanoparticles is lower than the nonmagnetic nanoparticles (*Al*_2_*O*_3_). The electrically conducting ferrofluid flow in the magnetic field was studied by Maria et al.^11^ over a curved stretching disk. They assume that ferrofluid velocity is a decreasing function of the volume fraction of the nanoparticle. Khan et al.^12^ analyzed the impact of heat flux and variable viscosity for heat transfer rate in the presence of magnetohydrodynamic ferrofluid flow. They gave the results that with the increasing volume fraction of nanoparticles, the rate of heat transfer and skin friction improves.

The most efficient nanoparticles are carbon nanotubes. Carbon nanotubes (CNT) are the tubular structures of carbons, also known as graphine sheets. Single Wall carbon nanotubes (SWCNT's) structure is a cylindrical shape which is composed of a single layer of graphene particles holding all the atoms at one place and has 0.5–1.5 nm diameter. MWCNT is a collection of graphene layer interconnected tubes of exponentially rising diameters. CNT are highly efficient nanotubes as they have six times better mechanical, physicochemical, and thermal properties as compared to other nanoparticles/nanotubes. CNTs play an important role in the field of optics, engineering, chemical production, material science, and microelectronic cooling. Hayat et al.^13^ discussed the Darcy–Forchheimer flow of water-based SWCNT and MWCNT over a rotating disk that is convectively heated. They reported that the velocity parameter of the fluid is higher with the increase in SWCNT and MWCNT volume fraction. The electromagnetohydrodynamic (EMHD) flow of kerosene oil and water base nanofluid flow over a stretching layer in the presence of thermal radiation was demonstrated by Zahir et al.^14^. They provided the results that a high density of CNTs in nanofluid possesses an increasing rate of heat transfer. The effect of MHD, suction/injection, and chemical reaction for the mass and heat transfer flow of water-based MWCNT and SWCNT over a porous vertical conic segment was explored by Sreedevi et al.^15^. They presented the knowledge that with an improvement in the volume fraction, the rate of heat transfer increases and that the increase in heat transfer rate in water-based MWCNT nanofluid is greater than that of SWCNT.

Owing to the advantages of the nanofluids, there is still some limitations/drawback of nanofluids, i.e., a single type of nanoparticles is not able to enhance all required properties of the base fluid. To overcome this problem, hybrid nanofluids have been introduced. Hybrid nanofluids are obtained by the insertion of two or more nanoparticles in the base fluid. In hybrid nanofluids, the physical and chemical properties of different materials combine simultaneously and provide a homogeneous mixture. These nanofluids have improved the impact of thermal conductivity and heat transfer rate which reduced the cost in industrial areas. Ghadikolaei and Gholinia^16^ worked on heat transfer and natural convection flow of hybrid nanofluid near a vertical porous stretching sheet. They showed the direct relation of nanofluid velocity with Grashof number and inverse relation with the magnetic field parameter. Iqbal et al.^17^ examined the collective impacts of thermal radiation and Hall current through a rotating vertical channel in the existence of transverse constant magnetic field and hybrid-nanofluid. Sajjadi et al.^18^ developed the Lattice Boltzmann (LBM) program for multi-wall carbon nanotubes to study the heat transfer impact of hybrid-nanofluid (*MWCNT*–*Fe*_3_*O*_4_ water). Their results demonstrate that by increasing the magnetic field, the heat transfer rate is reduced and this reduction is due to the Darcy number and increases because of the porosity parameter. An LBM numerical analysis used by Yuan et al.^19^ to analyze the hybrid nanofluid flow of the MHD *Ag*–*MgO*/*water* for heat transfer analysis across the tube. They noticed that applying *Ag*–*MgO* to water raises the rate of heat transfer and that the rate of heat transfer decreases by strengthening the Hartmanns number. During this analysis, they found that the production of entropy in the nanofluid *Al*_2_*O*_3_/*H*_2_*O* is smaller than the hybrid nanofluid *Cu*–*Al*_2_*O*_3_/*H*_2_*O*. Hayat and Nadeem^20^ have evaluated the flow of hybrid nanofluid (*Ag*–*CuO*/*water*) in the presence of thermal radiation over a linear stretching layer. They concluded that the hybrid nanofluid improves the heat transfer rate and distribution of temperature. The normal convection heat transfer flow of hybrid nanofluid was analyzed by Mehryan et al.^21^ in a complex porous T-shaped cavity with a cold upper and a hot bottom wall using a numerical technique. They argue that the magnetic field viscosity parameter decreases the rate of heat transfer inside the solid and liquid phases. The two-dimensional MHD flow and heat transfer of Hybrid nanoparticles *Cu*–*Al*_2_*O*_3_/*H*_2_*O* suspended in a micropolar dusty fluid were reviewed by Ghadikolaei et al.^22^ using the numerical technique RK of order 5. Mehrez and Cafsi^23^ reported under the prompt of magnetic field on the phenomena of induced convection flow of hybrid nanofluid (*Cu*–*Al*_2_*O*_3_/*H*_2_*O*). They showed that with the low Hartmann number, an increase in heat transfer rate is visible. In the last few years, several articles are published regarding the importance of hybrid nanofluids showing their importance in many fields of science and technology^24–33^.

The emission of electromagnetic waves from a heated surface to the surroundings in every direction is known as thermal radiation. Thermal radiations travel towards the absorption point with the speed of light. The thermal radiation and Hall current effect on mixed convection MHD rotating fluid flow in a porous vertical channel were combinedly studied by Singh and Pathak^34^. In an asymmetric channel that is tapered, Kothandapania and Prakash^35^ technically scrutinized the thermal radiation effect as well as the magnetic field on the peristaltic motion of the Williamson fluid. Theoretically, Narayana^36^ studied the thermal radiation and heat generation effect on mixed convection unstable flow through a porous and wavy vertical channel of the electrically conducting incompressible viscous fluid. Theoretically, Saswati and Rita^37^ calculate the thermal radiation and Hall current effect in a porous revolving channel on the MHD rotating flow of the elastic-viscous fluid. The effect of thermal radiation on the flow of viscous fluid in an asymmetric deformable horizontal porous channel was studied by Naveed et al.^38^.

There are many practical examples of fluid flow in the parallel deformable channels such as respiratory systems, coolant circulation, inter-body fluid transportation, aerospace engineering, and industrial cleaning procedures, etc. Yang et al.^39^ worked on MHD and heat transfer electroosmotic flow of a fluid in a microchannel of rectangular shape. Guillermo et al.^40^ combined the effects of entropy generation, thermal radiation, hydrodynamic slip, and MHD flow of a nanofluid in a porous horizontal microchannel. Zhao and Yang^41^ scrutinized the electroosmotic flow of the power-law (non-Newtonian) fluid in a cylindrical microchannel. Khan and Naz^42^ investigated the mass and heat transfer flow of three-dimensional second-grade fluid in a porous channel. They reported that the velocity of the fluid increases when there is no injection of fluid but at some point, velocity has an inverse relation with the suction parameter of the fluid. Rauf et al.^43^ discussed the thermally radiative mix convective nanofluid flow in a stretchable porous channel. Idowu et al.^44^ illustrated the thermal conductivity along with mass and heat transfer on the oscillatory MHD flow of the Jeffery fluid in a porous channel. Xinhui et al.^45^ proposed a research on the incompressible viscous flow of Newtonian fluid in an asymmetric porous channel. They investigated the rate of mass and heat transfer. Sheikholeslami^46^ studied the flow of water-based *CuO* nanofluid in a porous horizontal channel in the presence of a magnetic field. Aksoy and Pakdemirli^47^ obtained the approximate analytical solution of third-grade fluid flow in a parallel-plate porous channel. Bataineh et al.^48^ analyzed the rate of heat transfer and MHD flow of second-grade fluid in a channel having porous walls. They used the famous numerical technique RK4 and homotopy analysis method to get the graphical results of velocity and temperature parameters.

Inspiring from the above-referred literature review, it is noticed that the study of Hybrid nanofluid with *Fe*_2_*O*_3_, and SWCNT, MWCNTs through a horizontal parallel porous channel with the magnetic field is still missing and owning to its importance in many engineering and industrial applications, it is addressed in this article. Formation of hybrid nanofluid, using these kinds of particles (*Fe*_2_*O*_3_, single-wall carbon nanotubes and multi-wall carbon nanotubes) can be found in^24^. Due to the high-temperature phenomenon, the impact of thermal radiation is also assumed. Governing equations are modeled under different assumptions and are solved numerically by the shooting method. Runge–Kutta method of order four with Newton's method is used effectively for the graphical results. We believe that is study will uniquely contribute to predicting the significance of hybrid nanofluid through the horizontal porous channel.

## Mathematical formulation

**Figure 1:**
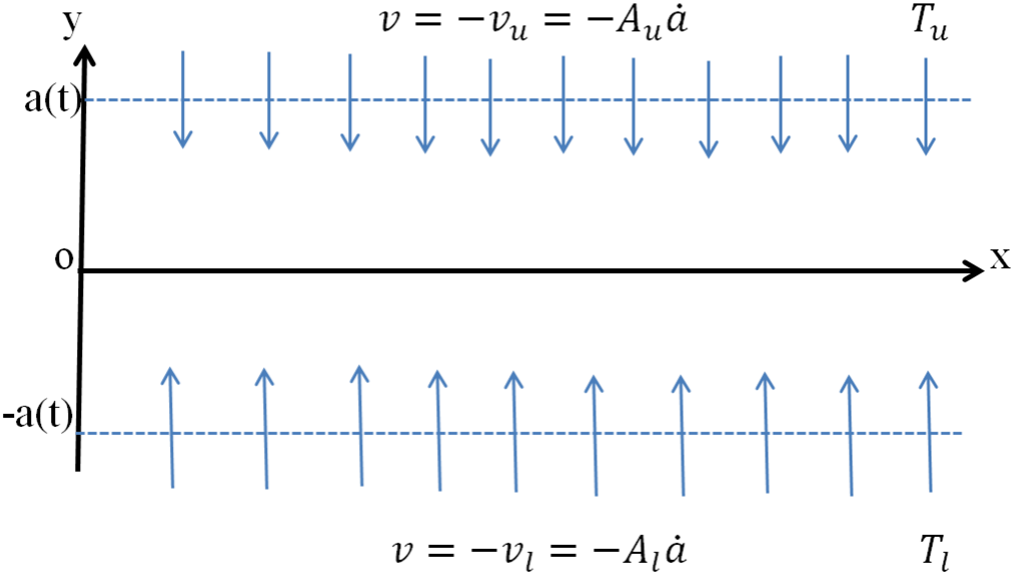
Geometry of the problem.

We considered a rectangular-shaped channel that is semi-infinite and protected by a dense pliable sheet at the leading edge. An electrically conducting unsteady fluid flows between these plates. Both walls have different porosity factors and squeeze or dilate with uniform rate. At the origin of the channel, the middle portion of the cylinder is set as shown in the Fig. 1. $T_{l}$ and $T_{u}$ are symbolized as the lower and upper wall temperature. Hybrid nanofluid (*CNT*–*Fe*_3_*O*_4_/*H*_2_*O*) flows through the channel which is viscid and incompressible. The physical flow properties of hybrid-nanofluid (*CNT*–*Fe*_3_*O*_4_/*H*_2_*O*) depends on time. Initially, the ferrous oxide nanoparticles (*Fe*_3_*O*_4_) of volume fraction $(\phi_{1} = 0.05 - 0.1)$ dissolved in the carrier fluid (*H*_2_*O*) which form the water-based Ferro-nanofluid (*Fe*_3_*O*_4_/*H*_2_*O*). These types of nanoparticles are deemed due to their magnetized properties. These have high influence for the electric and magnetic field. On the other hand, single and multi-wall carbon nanotubes have highest thermal conductivity. After that, hybrid nanofluid (*CNT*–*Fe*_3_*O*_4_/*H*_2_*O*) is formed by adding a different volume fraction of CNT $(\phi_{2} = 0.04)$ into the initially formed Ferro-fluid (*Fe*_3_*O*_4_/*H*_2_*O*). Also assumed that the walls of the rectangular channel are porous and fluid injection and suction take place due to dilation and squeezing of the walls. The porosity of the upper and lower walls was different and the middle is at the origin position of the channel. The mathematical representation of the mass, momentum and energy conservation of above explained model is determined as follows^24,29^:

1 $$ \frac{{\partial \tilde{v}}}{{\partial \tilde{y}}} + \frac{{\partial \tilde{u}}}{{\partial \tilde{x}}} = 0, $$

2 $$ \frac{{\partial \tilde{p}}}{{\partial \tilde{x}}} = \mu_{hnf} \left( {\frac{{\partial^{2} \tilde{u}}}{{\partial \tilde{x}^{2} }} + \frac{{\partial^{2} \tilde{u}}}{{\partial \tilde{y}^{2} }}} \right) - \rho_{hnf} \left( {\frac{{\partial \tilde{u}}}{{\partial \tilde{t}}} + \frac{{\partial \tilde{u}}}{{\partial \tilde{x}}}\tilde{u} + \frac{{\partial \tilde{u}}}{{\partial \tilde{y}}}\tilde{v}} \right) - \sigma_{hnf} B_{ \circ }^{2} \tilde{u}, $$

3 $$ \frac{{\partial \tilde{p}}}{{\partial \tilde{y}}} = \mu_{hnf} \left( {\frac{{\partial^{2} \tilde{v}}}{{\partial \tilde{x}^{2} }} + \frac{{\partial^{2} \tilde{v}}}{{\partial \tilde{y}^{2} }}} \right) - \rho_{hnf} \left( {\frac{{\partial \tilde{v}}}{{\partial \tilde{t}}} + \frac{{\partial \tilde{v}}}{{\partial \tilde{x}}}\tilde{u} + \frac{{\partial \tilde{v}}}{{\partial \tilde{y}}}\tilde{v}} \right), $$

4 $$ \frac{{\partial \tilde{T}}}{{\partial \tilde{t}}} + \frac{{\partial \tilde{T}}}{{\partial \tilde{x}}}\tilde{u} + \frac{{\partial \tilde{T}}}{{\partial \tilde{y}}}\tilde{v} = \delta_{hnf} \left( {\frac{{\partial^{2} \tilde{T}}}{{\partial \tilde{x}^{2} }} + \frac{{\partial^{2} \tilde{T}}}{{\partial \tilde{y}^{2} }}} \right) - \frac{1}{{\left( {\rho C_{p} } \right)_{hnf} }}\left( {\frac{\partial }{{\partial \tilde{x}}} + \frac{\partial }{{\partial \tilde{y}}}} \right)\,q_{rad} , $$

where

$$ q_{rad} = \frac{{4\mathop \sigma \limits^{*} \tilde{T}^{3} }}{{3a_{k} }}\frac{{\partial \tilde{T}}}{{\partial \tilde{y}}}, $$

subject to the boundary conditions

5 $$ \begin{array}{*{20}l} {{\text{at}}\;\tilde{y} = - a\left( {\tilde{t}} \right){:}} \hfill & {\quad \left( {\tilde{T} - \tilde{T}_{l} } \right) = 0,\;\,\tilde{v} = - \tilde{v}_{l} = - A_{l} \dot{a},\;\,\tilde{u} = 0,} \hfill \\ {{\text{at}}\;\tilde{y} = a\left( {\tilde{t}} \right){:}} \hfill & {\quad \left( {\tilde{T} - \tilde{T}_{u} } \right) = 0,\;\,\tilde{v} = - \tilde{v}_{u} = - A_{u} \dot{a},\;\,\tilde{u} = 0,} \hfill \\ \end{array} $$

where $\tilde{u}$ and $\tilde{v}$ are the velocity components in the $\tilde{x}$ and $\tilde{y}$ direction respectively, $\tilde{p}$ the pressure, $\mu_{hnf}$, $\sigma_{hnf}$, $\rho_{hnf}$ the dynamic viscosity, electric conductivity, density of hybrid-nanofluid, $B_{ \circ }$, $q_{rad}$, $\tilde{T}$, $C_{p}$, are the magnetic field strength, thermal radiation, the temperature of the fluid and the specific heat at constant pressure, $\sigma^{ * } \,$ is Boltzmann constant, $\tilde{T}_{l}$ and $\tilde{T}_{u}$ are the temperature of the lower and upper wall respectively, $a_{k}$ is for the mean absorbtion constant, $A_{l} ,\,A_{u}$ the permeability of lower and upper wall respectively.

Taking the partial derivatives of Eqs. (2) and (3) with respect to $\tilde{y}$ and $\tilde{x}$ respectively and eliminating the pressure gradient term, We get:

6 $$ \begin{aligned} & \mu_{hnf} \left( {\frac{{\partial^{3} \tilde{u}}}{{\partial \tilde{x}^{2} \partial \tilde{y}}} + \frac{{\partial^{3} \tilde{u}}}{{\partial \tilde{y}^{3} }}} \right) - \rho_{hnf} \left( {\frac{{\partial^{2} \tilde{u}}}{{\partial \tilde{t}\partial \tilde{y}}} + \tilde{u}\frac{{\partial^{2} \tilde{u}}}{{\partial \tilde{x}\partial \tilde{y}}} + \frac{{\partial \tilde{u}}}{{\partial \tilde{x}}}\frac{{\partial \tilde{u}}}{{\partial \tilde{y}}} + \tilde{v}\frac{{\partial^{2} \tilde{u}}}{{\partial \tilde{y}^{2} }} + \frac{{\partial \tilde{u}}}{{\partial \tilde{y}}}\frac{{\partial \tilde{v}}}{{\partial \tilde{y}}}} \right) - \sigma_{hnf} B_{ \circ }^{2} \frac{{\partial \tilde{u}}}{{\partial \tilde{y}}} \\ & \quad = \mu_{hnf} \left( {\frac{{\partial^{3} \tilde{v}}}{{\partial \tilde{y}^{2} \partial \tilde{x}}} + \frac{{\partial^{3} \tilde{v}}}{{\partial \tilde{x}^{3} }}} \right) - \rho_{hnf} \left( {\frac{{\partial^{2} \tilde{v}}}{{\partial \tilde{t}\partial \tilde{x}}} + \tilde{u}\frac{{\partial^{2} \tilde{v}}}{{\partial \tilde{x}^{2} }} + \frac{{\partial \tilde{v}}}{{\partial \tilde{x}}}\frac{{\partial \tilde{u}}}{{\partial \tilde{x}}} + \tilde{v}\frac{{\partial^{2} \tilde{v}}}{{\partial \tilde{x}\partial \tilde{y}}} + \frac{{\partial \tilde{v}}}{{\partial \tilde{y}}}\frac{{\partial \tilde{v}}}{{\partial \tilde{x}}}} \right) \\ \end{aligned} $$

In above equations, volumetric heat capacity $(\rho C_{p} )_{hnf}$, thermal diffusivity $\delta_{hnf} = \tfrac{{k_{hnf} }}{{(\rho C_{p} )_{hnf} }},$ thermal conductivity $k_{hnf}$ and electric conductivity $\sigma_{hnf}$ of the hybrid nanofluid are defined as^24,30^:

7 $$ \begin{aligned} & \frac{{\rho_{hnf} }}{{\mu_{hnf} }} = \frac{1}{{\nu_{hnf} }},\;\;\frac{{\sigma_{hnf} }}{{\sigma_{bf} }} = \frac{{\sigma_{CNT} + 2\sigma_{bf} - 2\phi_{2} (\sigma_{bf} - \sigma_{CNT} )}}{{\sigma_{CNT} + 2\sigma_{bf} + \phi_{2} (\sigma_{bf} - \sigma_{CNT} )}},\;\;\delta_{hnf} = \frac{{k_{hnf} }}{{\left( {\rho C_{p} } \right)_{hnf} }}, \\ & \frac{{\sigma_{bf} }}{{\sigma_{f} }} = \frac{{\sigma_{MS} + 2\sigma_{f} - 2\phi_{1} (\sigma_{f} - \sigma_{MS} )}}{{\sigma_{MS} + 2\sigma_{f} + \phi_{1} (\sigma_{f} - \sigma_{MS} )}},\;\;\mu_{hnf} = \frac{{\mu_{f} }}{{\left( {1 - \phi_{1} } \right)^{5/2} \left( {1 - \phi_{2} } \right)^{5/2} }}, \\ & \rho_{hnf} = \rho_{f} \left[ {(1 - \phi_{2} )\left( {1 - (1 - \frac{{\rho_{MS} }}{{\rho_{f} }})\phi_{1} } \right) + \phi_{2} \frac{{\rho_{CNT} }}{{\rho_{f} }}} \right], \\ & \frac{{\left( {\rho C_{p} } \right)_{hnf} }}{{\left( {\rho C_{p} } \right)_{f} }} = \left( {1 - \phi_{2} } \right)\,\left[ {1 - \left( {1 - \frac{{\left( {\rho C_{p} } \right)_{MS} }}{{\left( {\rho C_{p} } \right)_{f} }}} \right)\,\phi_{1} } \right] + \frac{{\left( {\rho C_{p} } \right)_{CNT} }}{{\left( {\rho C_{p} } \right)_{f} }}\phi_{2} , \\ & \frac{{k_{hnf} }}{{k_{bf} }} = \frac{{1 - \phi_{2} + 2\phi_{2} \tfrac{{k_{CNT} }}{{\left( {k_{CNT} - k_{bf} } \right)}}\ln \left( {\tfrac{{k_{CNT} + k_{bf} }}{{2k_{bf} }}} \right)}}{{1 - \phi_{2} + 2\phi_{2} \tfrac{{k_{bf} }}{{\left( {k_{CNT} - k_{bf} } \right)}}\ln \left( {\tfrac{{k_{CNT} + k_{bf} }}{{2k_{bf} }}} \right)}}, \\ & \frac{{k_{bf} }}{{k_{f} }} = \frac{{k_{MS} + \left( {m - 1} \right)\,k_{f} - \left( {m - 1} \right)\,\phi_{1} \left( {k_{f} - k_{MS} } \right)}}{{k_{MS} + \left( {m - 1} \right)\,k_{f} + \phi_{1} \left( {k_{f} - k_{MS} } \right)}}, \\ \end{aligned} $$

In the above expression (7), $k_{f}$ and $k_{bf}$ are respectively the thermal conductivity of base fluid *H*_2_*O* and *Fe*_2_*O*_3_-nanofluid. Here, $m$ must be chosen 3 for the spherical nanoparticles. Also, and $k_{CNT}$ signifies the thermal conductivity of *Fe*_2_*O*_3_ and CNTs respectively. $\varphi_{1} ,\,\,\varphi_{2} ,\,\left( {C_{p} } \right)_{CNT} ,\,\left( \rho \right)_{CNT} ,\,(C_{p} )_{f} ,\,\rho_{f} ,\,\mu_{f}$ are respectively indicates the volume fraction of *Fe*_2_*O*_3_, volume fraction of CNTs, specific heat at constant pressure of CNTs, density of CNTs, specific heat at constant pressure of base fluid, density of base fluid, and dynamic viscosity of the carrier fluid. The electric conductivity of *Fe*_2_*O*_3_-nanofluid and base fluid are represented by $\sigma_{bf}$ and $\sigma_{f}$ respectively. Table 1 shows the thermal and physical properties of carrier fluid (*H*_2_*O*), nanoparticles *Al*_2_*O*_3_, and carbon nanotubes.

**Table 1:** Experimental upshots of traits of *H*_2_*O*, *Fe*_3_*O*_4_, SWCNT and MWCNT.

Material	*H*_2_*O* (f)	*Fe*_3_*O*_4_ (MS)	SWCNT	MWCNT
*ρ* (kg m^−3^)	997.1	5200	2600	1600
*C*_*p*_ (J kg^−1^ K^−1^)	4179	670	425	796
*k* (W m^−1^ K^−1^)	0.613	6	6600	3000
*σ* (s m^−1^)	5.5 × 10^−6^	0.74 × 10^6^	10^6^	10^7^

Applying the similarity transformation^24^;

8 $$ \xi = \frac{{\tilde{y}}}{{a\left( {\tilde{t}} \right)}},\,\;\;\tilde{u} = \frac{{\nu_{f} \tilde{x}\overline{F}_{\xi } \left( {\xi ,\,\tilde{t}} \right)}}{{a^{2} \left( {\tilde{t}} \right)}},\,\;\;\tilde{v} = - \frac{{\nu_{f} \overline{F} \left( {\xi ,\,\tilde{t}} \right)}}{{a\left( {\tilde{t}} \right)}},\,\;\;T(\xi ) = \frac{{\tilde{T} - \tilde{T}_{u} }}{{\tilde{T}_{l} - \tilde{T}_{u} }}. $$

The similarity transformation satisfies the continuity Eq. (1) identically, while the momentum Eq. (6) and energy Eq. (4) got the following non-dimensional form;

9 $$ \overline{F}_{\xi \xi \xi \xi } + \frac{{\nu_{f} }}{{\nu_{hnf} }}\left( {\alpha \left( {3\overline{F}_{\xi \xi } + \xi \overline{F}_{\xi \xi \xi } } \right) - \overline{F}_{\xi } \overline{F}_{\xi \xi } + \overline{F}\overline{F}_{\xi \xi \xi } } \right) - \overline{F}_{\xi \xi } MD_{1} D_{2} - \frac{{a^{2} }}{{\nu_{hnf} }}\overline{F}_{{\xi \xi \tilde{t}}} = 0, $$

10 $$ - \frac{{k_{hnf} }}{{k_{f} }}T^{\prime\prime} - \Pr \frac{{\left( {\rho C_{p} } \right)_{hnf} }}{{\left( {\rho C_{p} } \right)_{f} }}\left( {\alpha \xi + FR} \right)\,T^{\prime} + Rd\left[ {\begin{array}{*{20}c} {\left( {1 + \left( {T_{r} - 1} \right)T(\xi )} \right)^{3} T^{\prime\prime}} \\ { + 3\left( {1 + \left( {T_{r} - 1} \right)T(\xi )} \right)^{2} T^{\prime 2} \left( {T_{r} - 1} \right)} \\ \end{array} } \right] = 0. $$

In the above equations, the wall’s deformation rate is $\alpha = a\dot{a}/\nu_{f}$ and its value is considered to be positive for the dilating channel. Xinhui et al.^45^ suggested that for the uniformity of $\alpha \,$ in time, a similar solution w.r.t. both time and space can be accomplished by choosing $\alpha \,$ a constant and it leads $\overline{F}_{{\xi \xi \tilde{t}}} = 0.$ To figure out this condition, the wall’s deformation rate (expansion ratio) $\alpha \,$ must be prescribed by the channel's initial height.

11 $$ \overline{F}_{\xi \xi \xi \xi } + \frac{{\nu_{f} }}{{\nu_{hnf} }}\left( {\alpha \left( {3\overline{F}_{\xi \xi } + \xi \overline{F}_{\xi \xi \xi } } \right) - \overline{F}_{\xi } \overline{F}_{\xi \xi } + \overline{F}\overline{F}_{\xi \xi \xi } } \right) - \overline{F}_{\xi \xi } MD_{1} D_{2} = 0. $$

The boundary conditions are:

12 $$ \begin{array}{*{20}l} {{\text{at}}\;\,\tilde{y} = - a\left( {\tilde{t}} \right){:}} \hfill & {\quad \overline{F}\left( {\xi ,\tilde{t}} \right)|_{\xi = - 1} = R_{l} ,\;\;\overline{F}_{\xi } \left( {\xi ,\tilde{t}} \right)|_{\xi = - 1} = 0,\;\;T\left( {a^{ - 1} \left( {\tilde{t}} \right)\,\tilde{y}} \right)|_{\xi = - 1} = 1,} \hfill \\ {{\text{at}}\;\,\tilde{y} = a\left( {\tilde{t}} \right){:}} \hfill & {\quad T\left( {a^{ - 1} \left( {\tilde{t}} \right)\tilde{y}} \right)_{\xi = 1} = 0,\;\;\overline{F}\left( {\xi ,\tilde{t}} \right)|_{\xi = 1} = R,\;\;\overline{F}_{\xi } \left( {\xi ,\tilde{t}} \right)|_{\xi = 1} = 0,} \hfill \\ \end{array} $$

In Eq. (12), $R_{l} = \tfrac{{\tilde{\nu }_{l} a}}{{\nu_{f} }}$ and $R = \tfrac{{\tilde{\nu }_{u} a}}{{\nu_{f} }}$ are respectively the Reynolds number with reference to the top and bottom wall of the horizontal channel. These are negative for suction case whereas positive for the injection case. Implementing the following scale variable for the further simplification of the governing Eq. (11) and boundary conditions (12).

13 $$ \overline{u} = \dot{a}^{ - 1} u,\,\;\;\overline{v} = \dot{a}^{ - 1} v,\,\;\;\overline{x} = \dot{a}^{ - 1} x,\,\;\;\overline{F} = FR, $$

This transformation (13) yields the following equation;

14 $$ F^{{\left( {iv} \right)}} + \lambda_{1} \left\{ {\alpha (3F^{\prime\prime} + \xi F^{\prime\prime\prime}) - R(F^{\prime}F^{\prime\prime} - FF^{\prime\prime\prime})} \right\} - F^{\prime\prime}RMD_{1} D_{2} = 0, $$

and the temperature Eq. (10) becomes;

15 $$ \lambda_{3} T^{\prime\prime} + \Pr \lambda_{2} \left( {\alpha \xi + FR} \right)\,T^{\prime} - Rd\left[ {\begin{array}{*{20}c} {\left( {1 + \left( {T_{r} - 1} \right)T(\xi )} \right)^{3} T^{\prime\prime}} \\ { + 3\left( {1 + \left( {T_{r} - 1} \right)T(\xi )} \right)^{2} T^{\prime 2} \left( {T_{r} - 1} \right),} \\ \end{array} } \right] = 0. $$

where

16 $$ \begin{aligned} M & = \frac{{\sigma_{f} B_{ \circ }^{2} a^{2} }}{{\rho_{f} \upsilon_{f} }},\,\;\;D_{2} = \left[ {\left( {1 - \phi_{1} } \right)^{5/2} \left( {1 - \phi_{2} } \right)^{5/2} } \right],\,\;\;T_{r} = \frac{{\tilde{T}_{l} }}{{\tilde{T}_{r} }},\,\;\;\Pr = \frac{{\delta_{f} }}{{\upsilon_{f} }}, \\ Rd & = \frac{{4\mathop \sigma \limits^{ * } \tilde{T}_{u}^{3} }}{{3a_{k} k_{f} }},\,\;\;D_{1} = \frac{{\sigma_{CNT} + 2\sigma_{bf} - 2\phi_{2} \left( {\sigma_{bf} - \sigma_{CNT} } \right)}}{{\sigma_{CNT} + 2\sigma_{bf} + \phi_{2} \left( {\sigma_{bf} - \sigma_{CNT} } \right)}} \times \frac{{\sigma_{MS} + 2\sigma_{f} - 2\phi_{1} \left( {\sigma_{f} - \sigma_{MS} } \right)}}{{\sigma_{MS} + 2\sigma_{f} + \phi_{1} \left( {\sigma_{f} - \sigma_{MS} } \right)}}, \\ \lambda_{1} & = \frac{{\upsilon_{f} }}{{\upsilon_{hnf} }} = \left( {1 - \phi_{1} } \right)^{5/2} \left( {1 - \phi_{2} } \right)^{5/2} \left[ {\left\{ {1 - \left( {1 - \frac{{\rho_{s} }}{{\rho_{f} }}} \right)\,\phi_{1} } \right\}\left( {1 - \phi_{2} } \right) + \frac{{\rho_{CNT} }}{{\rho_{f} }}\phi_{2} } \right], \\ \lambda_{3} & = \frac{{k_{hnf} }}{{k_{f} }},\,\;\;\lambda_{2} = \frac{{\left( {\rho C_{p} } \right)_{hnf} }}{{\left( {\rho C_{p} } \right)_{f} }}, \\ \end{aligned} $$

The transformed form of auxiliary conditions is:

17 $$ \begin{array}{*{20}l} {{\text{at}}\;\,\tilde{y} = - a\left( {\tilde{t}} \right){:}} \hfill & {\quad F\left( {a^{ - 1} \left( {\tilde{t}} \right)\tilde{y}} \right) = A,\;\;F^{\prime}\left( {a^{ - 1} \left( {\tilde{t}} \right)\tilde{y}} \right) = 0,\;\;T\left( {a^{ - 1} \left( {\tilde{t}} \right)\tilde{y}} \right) = 1,} \hfill \\ {{\text{at}}\;\,\tilde{y} = a\left( {\tilde{t}} \right){:}} \hfill & {\quad F\left( {a^{ - 1} \left( {\tilde{t}} \right)\tilde{y}} \right) = 1,\;\;F^{\prime}\left( {a^{ - 1} \left( {\tilde{t}} \right)\tilde{y}} \right) = 0,\;\;T\left( {a^{ - 1} \left( {\tilde{t}} \right)\tilde{y}} \right) = 0,} \hfill \\ \end{array} $$

here, the permeability parameter is symbolized as $A = \nu_{l} /\nu_{u} .$

The local Nusselt number, (rate of heat transfer) and skin friction are defined as:

$$ C_{f} = \frac{{a(t)\tau_{w} }}{{\rho_{hnf} \tilde{v}_{l}^{2} x}}, $$

with

$$ \tau_{w} = \mu_{hnf} \left( {\frac{\partial u}{{\partial y}}} \right)_{y = \pm a(t)} , $$

18 $$ Nu = - \frac{{ak_{f}^{ - 1} }}{{\left( {\tilde{T}_{l} - \tilde{T}_{u} } \right)}}\left( {k_{hnf} \frac{{\partial \tilde{T}}}{\partial y} + q_{r} } \right)_{y = \pm a(t)} , $$

The defined transformation can convert the above expression into dimensionless form. The local Nusselt number for the upper and lower wall of the channel are:

19 $$ \begin{array}{*{20}l} {C_{f} R_{l}^{2} = \frac{{F^{\prime\prime}( - 1)}}{{D_{2} \left[ {(1 - \phi_{2} )\left( {1 - \left( {1 - \tfrac{{\rho_{MS} }}{{\rho_{f} }}} \right)\phi_{1} } \right) + \phi_{2} \tfrac{{\rho_{CNT} }}{{\rho_{f} }}} \right]}},} \hfill & {\quad Nu_{lower} = - T^{\prime}\left( { - 1} \right)\,\left( {\lambda_{3} + Rd\left( {1 + \left( {T_{r} - 1} \right)\,T\left( { - 1} \right)} \right)^{3} } \right),} \hfill \\ {_{f} R_{u}^{2} = \frac{{F^{\prime\prime}(1)}}{{D_{2} \left[ {(1 - \phi_{2} )\left( {1 - \left( {1 - \tfrac{{\rho_{MS} }}{{\rho_{f} }}} \right)\phi_{1} } \right) + \phi_{2} \tfrac{{\rho_{CNT} }}{{\rho_{f} }}} \right]}},} \hfill & {\quad Nu_{upper} = - T^{\prime}\left( 1 \right)\,\left( {\lambda_{3} + Rd\left( {1 + \left( {T_{r} - 1} \right)\,T\left( { - 1} \right)} \right)^{3} } \right).} \hfill \\ \end{array} $$

## Solution methodology

To solve the governed ODEs in above section, a well-known numerical technique Runge–Kutta method of order four (RK4) along with shooting technique has been used. This numerical technique is much simpler as compared to the FVM (finite volume method), FEM (finite element method) or LBM (lattice Boltzman method). It has less computational cost. With the use of RK-4 and Newton's method, its results are effective and more reliable than any other analytical technique. In first step, highly coupled and nonlinear ODEs Eqs. (14) and (15) has been reduced to non-linear first order ODEs by assuming:

$$ Q_{1} = F,\;Q_{2} = F^{\prime},\;Q_{3} = F^{\prime\prime},\;Q_{4} = F^{\prime\prime\prime},\;Q_{5} = T,\;Q_{6} = T^{\prime} $$

So the transformed first order equations are:

20 $$ \begin{aligned} Q_{1}^{\prime } & = Q_{2} , \\ Q_{2}^{\prime } & = Q_{3} , \\ Q_{3}^{\prime } & = Q_{4} , \\ Q_{4}^{\prime } & = \lambda_{1} \left\{ {R\left( {Q_{2} Q_{3} - Q_{1} Q_{4} } \right) - \alpha \left( {3Q_{3} + \xi Q_{4} } \right)} \right\} + Q_{3} RMD\left[ {\left( {1 - \phi_{1} } \right)^{5/2} \left( {1 - \phi_{2} } \right)^{5/2} } \right], \\ Q_{5}^{\prime } & = Q_{6} , \\ Q_{6}^{\prime } & = \frac{{\Pr \lambda_{2} \left( {\alpha \xi + Q_{1} R} \right)\,Q_{6} - 3Rd\left\{ {1 + \left( {T_{r} - 1} \right)Q_{5} } \right\}^{2} Q_{6}^{2} \left( {T_{r} - 1} \right)}}{{Rd\left\{ {1 + \left( {T_{r} - 1} \right)Q_{5} } \right\}^{3} - \tfrac{{k_{hnf} }}{{k_{f} }}}}, \\ \end{aligned} $$

and the initial conditions are

21 $$ Q_{1} = A,\;\;Q_{2} = 0,\;\;Q_{5} = 1, $$

22 $$ Q_{3} = E,\;\;Q_{4} = G,\;\;Q_{6} = H, $$

here, *E, G* and *H* are the assumed initial guesses. The above system of equations is numerically integrated by RK-4 method and initial guesses are modified by utilizing Newton's method. Programming is done on MATLAB. All the results are computed with the tolerance of 10^−5^. The code is verified with numerically comparing the results of $F^{\prime\prime}(1)$, and it is found in a good agreement as shown in Table 2.

**Table 2:** Comparison of numerical results of *F*″(1) when *ϕ*_1_ = *ϕ*_2_ = *α* = *θ* = 0.

M	S	Taseer et al.^49^	Present
0	0.5	4.713303	4.713254
1		4.739017	4.739148
2		4.820251	4.820361
3		4.396487	4.396271
2	0	1.842447	1.842331
	0.3	3.653695	3.653601
	0.6	5.391248	5.391148
	1	7.593426	7.593006

## Results and discussions

This segment's main concern is to precisely show up the physical importance of the graphical simulations. The fluid flow with the transfer of heat characteristics of a hybrid nanofluid (*CNT*–*Fe*_3_*O*_4_/*H*_2_*O*) was observed through a porous permeable channel whose walls often display partitioning motion. In addition, the main objective is to envision the effect on velocity and temperature distribution of various physical parameters such as Reynolds number $R,$ rate of wall deformation $\alpha ,$ solid volume fraction $(0.005 \le \varphi_{2} \le 0.06),$ magnetic parameter $M,$ temperature ratio parameter $Tr,$ thermal radiation parameter $Rd,$ and porosity parameter $A.$ The physical and thermal characteristics of base fluid (*H*_2_*O*), Ferro-oxide $(Fe_{3} O_{4} ),$ and CNTs have been displayed in Table 1. Therefore, since this analysis includes water as a carrier fluid, it is presumed that the Prandtl number is equal to 6.2.

**Figure 2:**
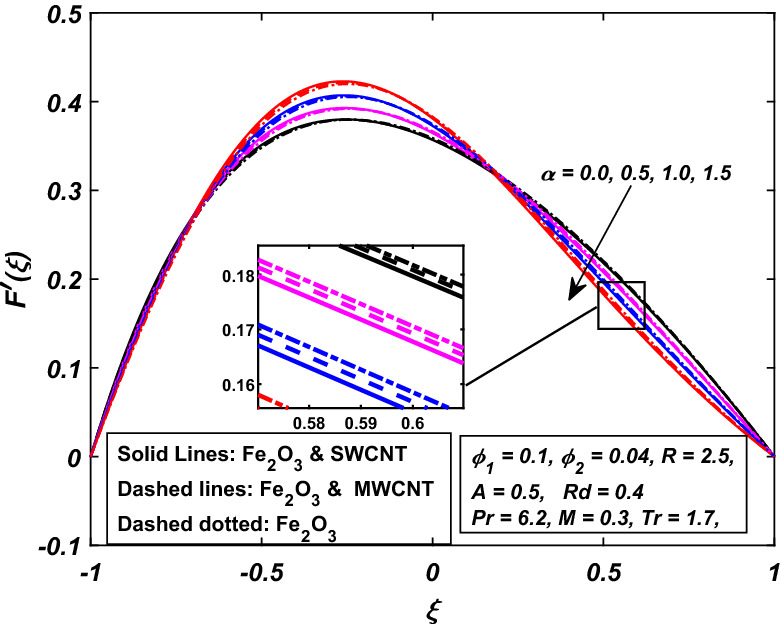
Influence of *α* on $F^{\prime}\left( \xi \right)$.

**Figure 3:**
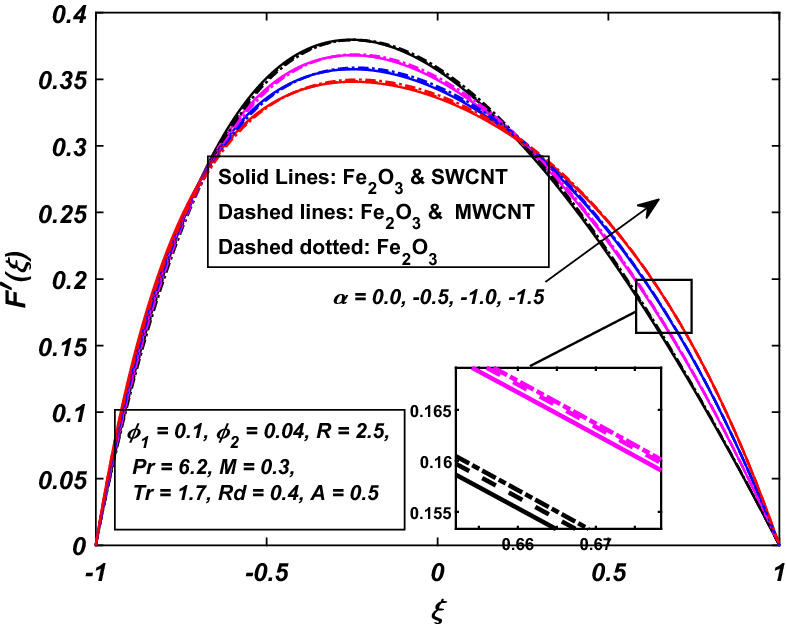
Influence of − *α* on $F^{\prime}\left( \xi \right)$.

Before starting to visualize the figures for the different parameters, it is necessary to know that $\alpha \,$ symbolize the wall deformation rate which is positive for the dilating wall channel whereas it is negative for the squeezing channel. Similarly, $R\,$ is the permeation Reynolds number which is negative for the injection case and positive for the suction. Figures 2 and 3 are designed to see the velocity conduct against the rising wall deformation rate $\alpha \,$ while dilating and contracting channel accompanied by injection $R > 0.$ Figure 2 indicates that when the walls undergo injection and are allowed to expand $(\alpha > 0)$, a vacant region is created near the walls, primarily because of the dilation of the channel. The neighboring layers of fluid are then moved inwards to fill this area and, as a result, a decrement in the speed of the fluid is observed, which slows down the rate of flow along the walls. On contrary, the velocity of the fluid increases with the absolute values of $\alpha \,$ in the middle of the channel, and hence, the conservation of momentum has been maintained. It is also noted that decrement in the velocity of the fluid is more prominent in the case of hybrid-nanofluid (*Fe*_2_*O*_3_–*SWCNTMWCNT*–*H*_2_*O*) as compared to simple nanofluid (*Fe*_2_*O*_3_–*H*_2_*O*). Furthermore, while contracting channel accomplished by injection. Figure 3 shows the neighboring fluid layers suppress near the channel's walls, and hence, an increment in velocity takes place, which speeds up the flow rate along the walls but the speed of fluid slows down in the middle of the channel. Here, the hybrid-nanofluid moves more quickly than the nanofluid.

**Figure 4:**
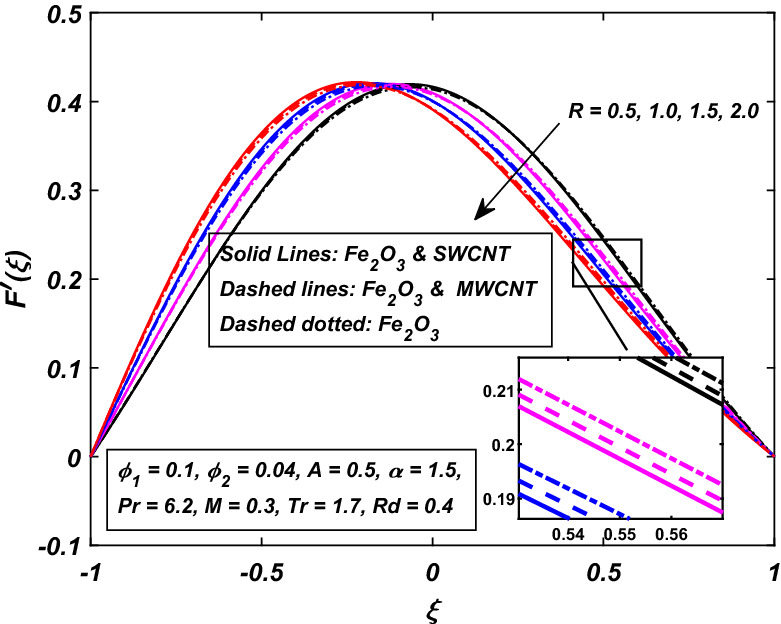
Influence of *R* on $F^{\prime}\left( \xi \right)$.

**Figure 5:**
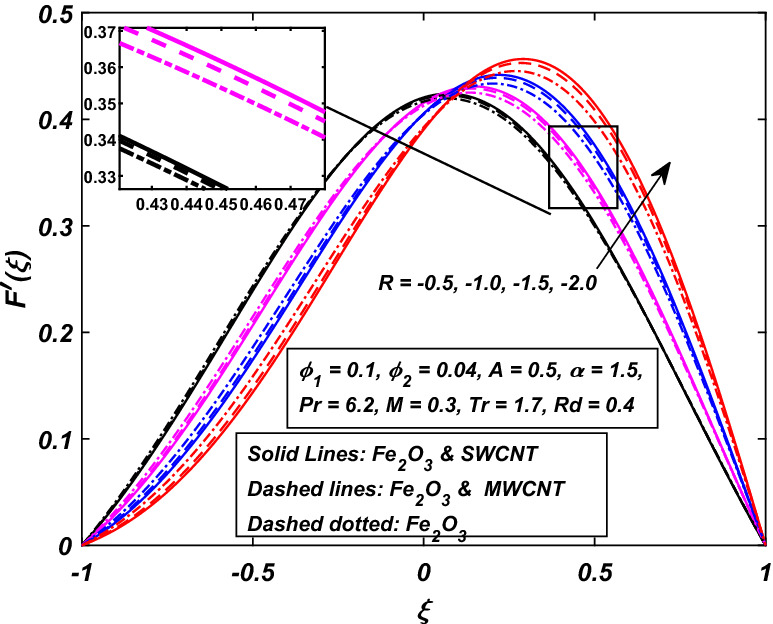
Influence of − *R* on $F^{\prime}\left( \xi \right)$.

Figures 4 and 5 were painted to investigate the velocity actions when the walls are embraced or partitioned with injection or suction together. Figure 4 shows the velocity action when the fluid is injected at the walls along with the channel’s expanding motion. The subjacent area of the channel shows that the velocity of the fluid slows down strongly with the increasing value of absolute $R.$ Figure 4 also indicates that the velocity of (*CNT*–*Fe*_3_*O*_4_/*H*_2_*O*) hybrid nanofluid in the channel’s top portion is dominant as compared to the (*Fe*_3_*O*_4_/*H*_2_*O*) nanofluid. MWCNTs also hold superiority in comparison with SWCNTs, just in the top portion of the channel. Also, Fig. 5 is represented to explain the situation when the dilating action of the walls occurs along with suction. The channel’s subjacent area shows an increase in velocity of the fluid with the increasing absolute $R\,$ likely because of the porosity of the walls $A = \tilde{v}_{l} /\tilde{v}_{u} .$ Accordingly, the term $A = 0.5\,$ suggests that the suction regulates the flow activity in the channel’s top portion and that increased fluid flow was observed. It was clearly shown from Fig. 5 that the velocity of (*Fe*_3_*O*_4_/*H*_2_*O*) nanofluid in the channel’s top portion is dominant as compared to the (*CNT*–*Fe*_3_*O*_4_/*H*_2_*O*) hybrid nanofluid. Single wall carbon nanotubes (SWCNTs) also hold superiority in comparison with MWCNTs, just in the channel’s top portion.

**Figure 6:**
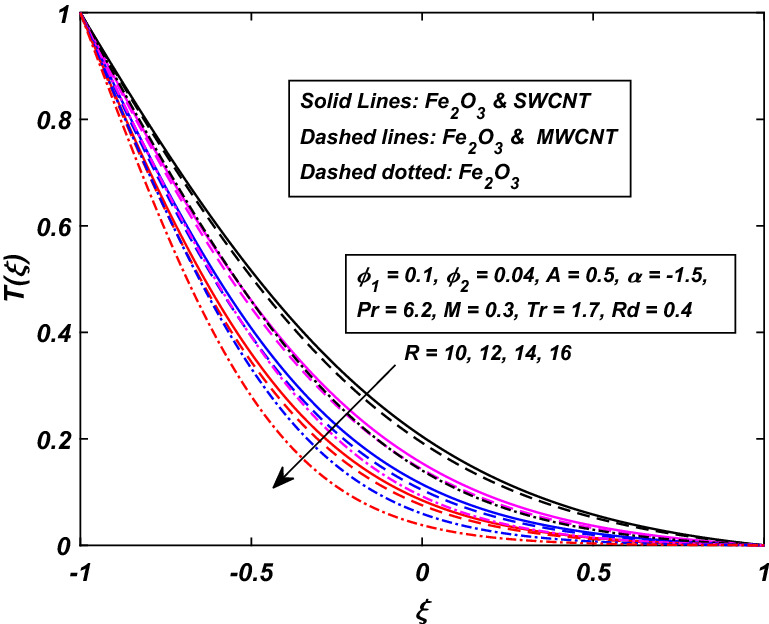
Influence of *R* on *T*(*ξ*).

**Figure 7:**
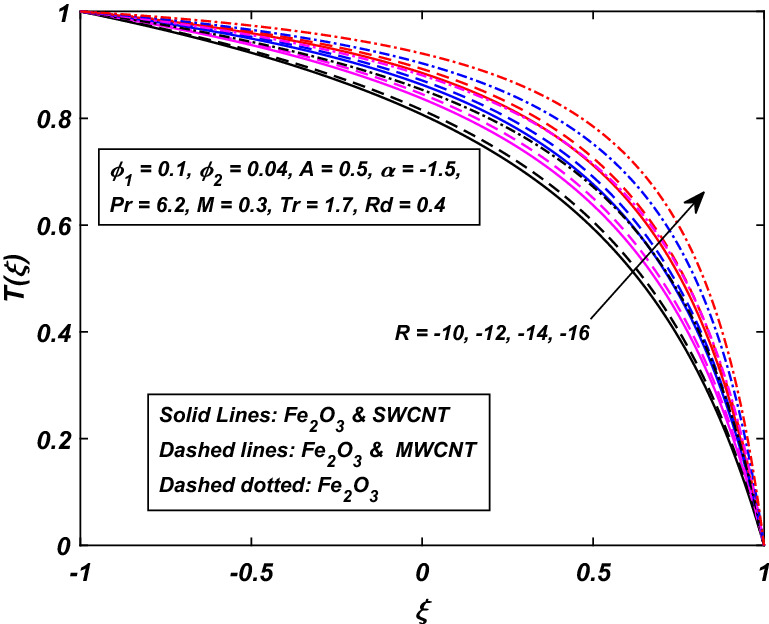
Influence of − *R* on *T*(*ξ*).

Figures 6 and 7 are drawn to describe the temperature behavior when the walls are squeezing with injection or suction together. Figure 6 shows the temperature behavior of fluid when the fluid’s injection at the walls along with the channel’s squeezing motion. It is found that the temperature demolished for the higher injection rate $R\,$. Due to more injection of nanofluid, the collision between the particles increases which ultimately enhances the temperature of the fluid. Interestingly, the gap between the hybrid-nanofluid and simple nanofluid is too wide, which indicates that a decline in the temperature of nanofluid (*Fe*_2_*O*_3_) is eminent, while Fig. 7 represents the situation when the dilating $(\alpha > 0)\,$ action of the walls occurs along with suction $(R < 0).$ We noticed a reverse relation as compared to injection case $(R > 0).$ When the walls are allowed to contract $(\alpha < 0)\,$ and fluid is sucked from the walls, the temperature of the fluid is enhanced. Here, nanofluid (*Fe*_3_*O*_4_) gains more temperature over the hybrid-nanofluid.

**Figure 8:**
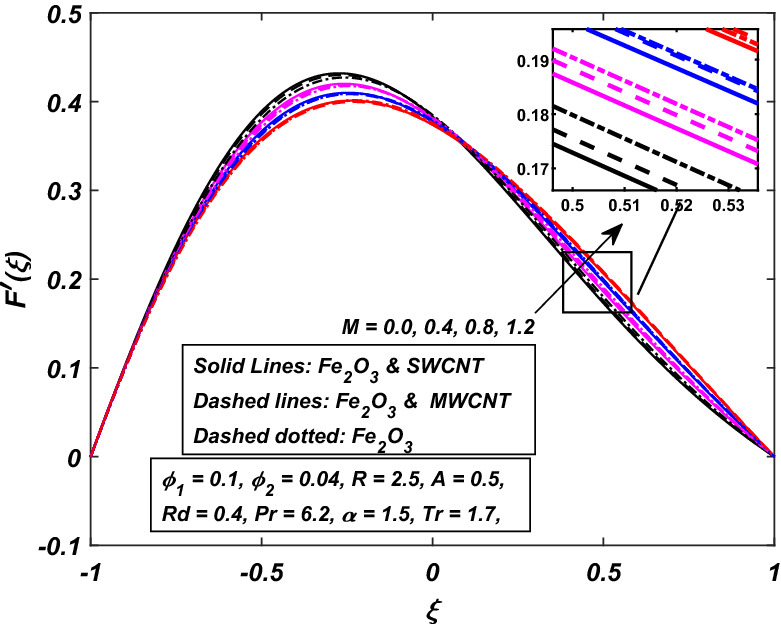
Influence of *M* on $F^{\prime}\left( \xi \right)$.

**Figure 9:**
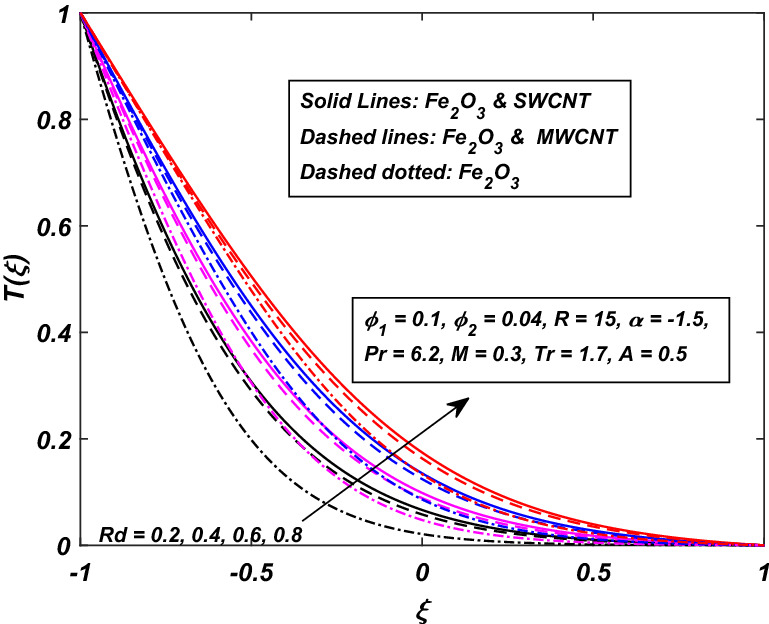
Influence of *Rd* on *T*(*ξ*).

**Figure 10:**
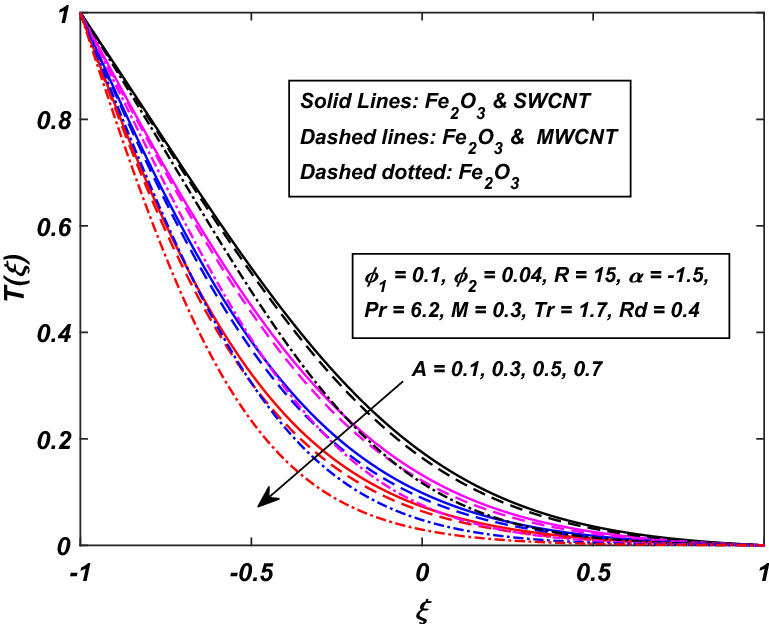
Influence of *A* on *T*(*ξ*).

**Figure 11:**
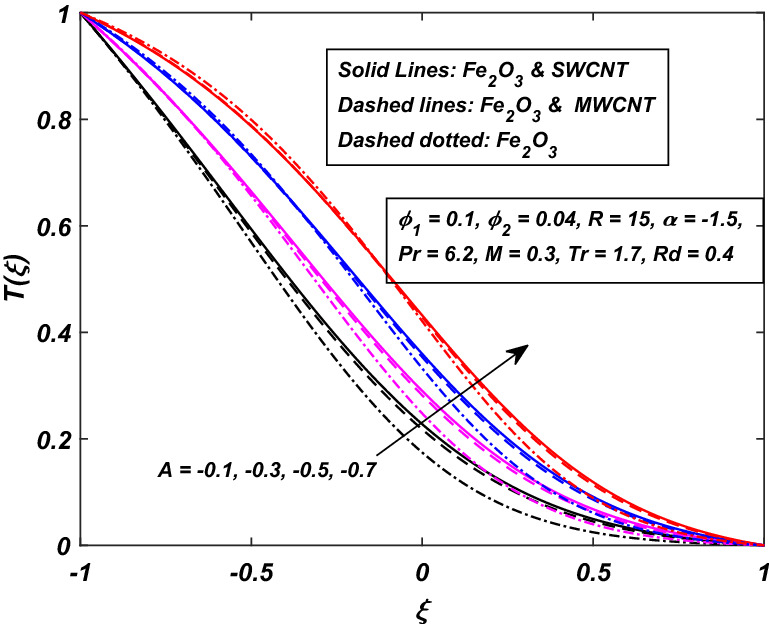
Influence of − *A* on *T*(*ξ*).

**Figure 12:**
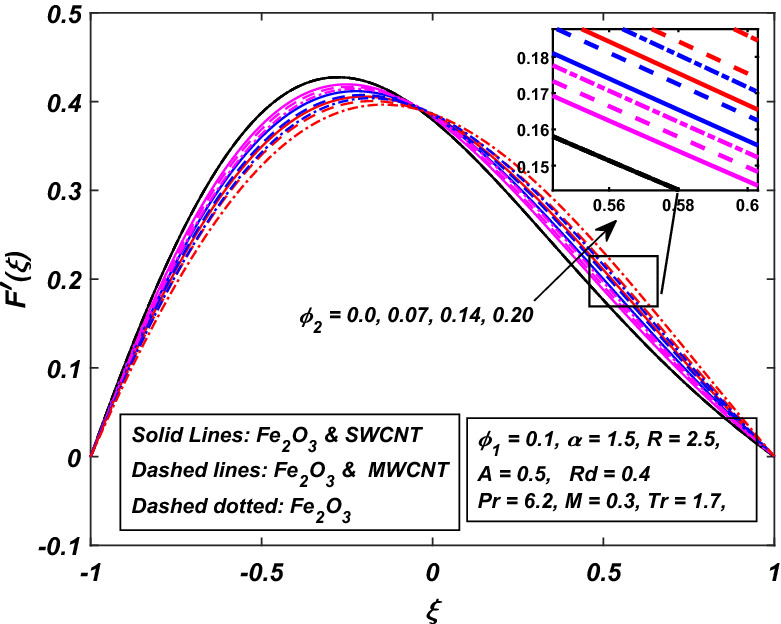
Influence of *φ*_2_ on $F^{\prime}\left( \xi \right)$.

**Figure 13:**
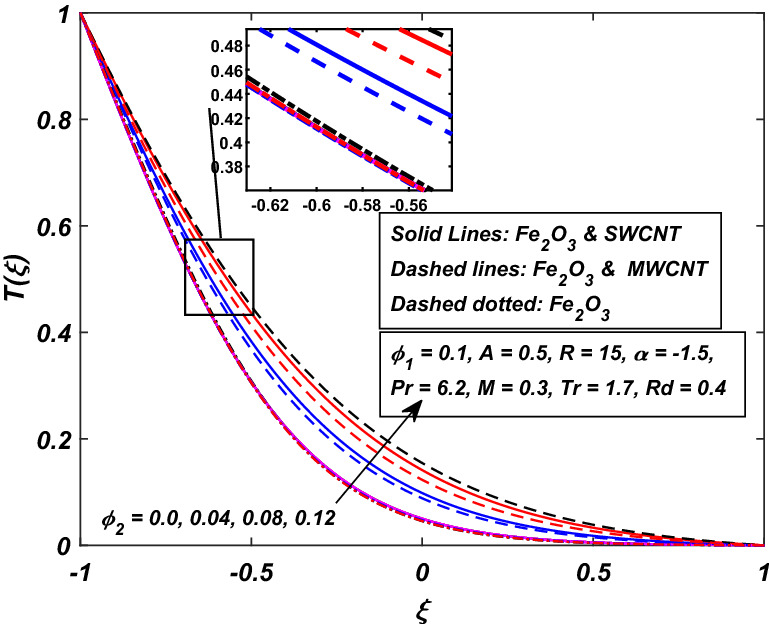
Influence of *φ*_2_ on *T*(*ξ*).

The impact of the magnetic parameter $M\,$ over the velocity profile $F^{\prime}(\xi )\,$ when both $\alpha \,$ and $R\,$ are positive, i.e. channel’s walls are expanding with the injection of fluid, is displayed in Fig. 8. By raising the magnetic field strength $M,$ the velocity of the fluid decreases near the lower portion while it increases on the other one. The utilization of a transverse magnetic field to an electrically conducting fluid produces a drag-like Lorentz force. The fluid velocity is slowed by this force. This is especially useful in magnetic materials processing operations that use a static transverse uniform magnetic field because it allows for precise flow field control. It is also observed that the Ferrofluid (*Fe*_3_*O*_4_/*H*_2_*O*) shows primacy in speed as compared to the (*CNT*–*Fe*_3_*O*_4_/*H*_2_*O*) hybrid-nanofluid. The parameter thermal radiation *Rd* is very influential for the increment of temperature as illustrated in Fig. 9. It is clear that the temperature, for the injection and contraction of the walls of the channel, rises significantly. The behavior close to the upper wall is the same for injecting and expanding cases, i.e. a higher temperature with rising *Rd* With the increasing values of the thermal radiation parameter *Rd*, the mean coefficient of absorption decreases, and as a result, a rise in the fluid temperature is expected. Here again, in comparison, a rise in temperature is more prominent in hybrid-nanofluid, especially for SWCNTs. Figure 10 assists in visualizing temperature variations under the power of porosity variable $A\,$ with the simultaneous squeezing or injection scenario. The graph represents the fact that an increase in the porosity parameter $A\,$ of the channel causes a clear decrement in the temperature profile. It is also clear from Fig. 10 that the hybrid-nanofluid poses dominant behavior in temperature relative to the ferrofluid. Figure 11 is sketched to assist in visualizing the temperature variations under the effect of the absolute parameter of porosity $A\,$ along with the squeezing/injection scenario. The graph carries higher values of temperature with an increase in the value of absolute $A\,$. This is because of the predominance of temperature at the channel’s lower wall, so a very less amount of temperature difference was observed near the upper wall in the region. Also, it was recorded that the region, located near the channel’s bottom wall, shows an increase in temperature profile, which is because of the fluid with higher thermal energy induced from the channel’s lower extremity. When it goes on, this injected fluid slowly experiences a loss of energy and eventually exits, the upper part of the channel, with the least possible temperature. In addition, Fig. 11 display that the hybrid nanofluid poses dominance in temperature relative to Ferrofluid. Figures 12 and 13 are plotted to test the effect of volume fraction $\varphi_{2}$ of nano-meter-sized particles of SWCNTs and MWCNTs. By raising the number of CNTs $\varphi_{2}$ in the base fluid, the subjacent part of the channel shows higher values of velocity and temperature respectively in Figs. 12 and 13. In 12 Hybrid-nanofluid’s (*CNT*–*Fe*_3_*O*_4_/*H*_2_*O*) velocity tends to have lower values as compared to the ferrofluids whereas in 13 Hybrid-nanofluid’s (*CNT*–*Fe*_3_*O*_4_/*H*_2_*O*) temperature is more prominent.

**Figure 14:**
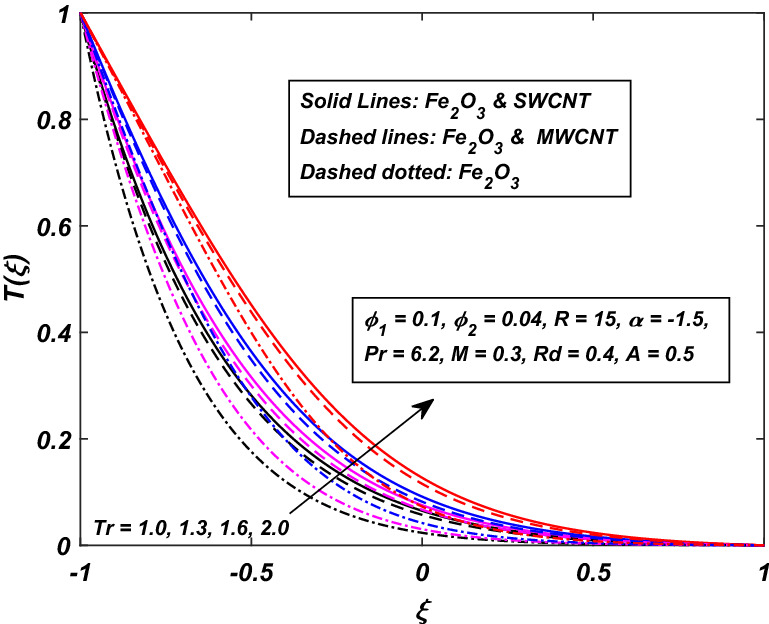
Influence of *Tr* on *T*(*ξ*).

**Figure 15:**
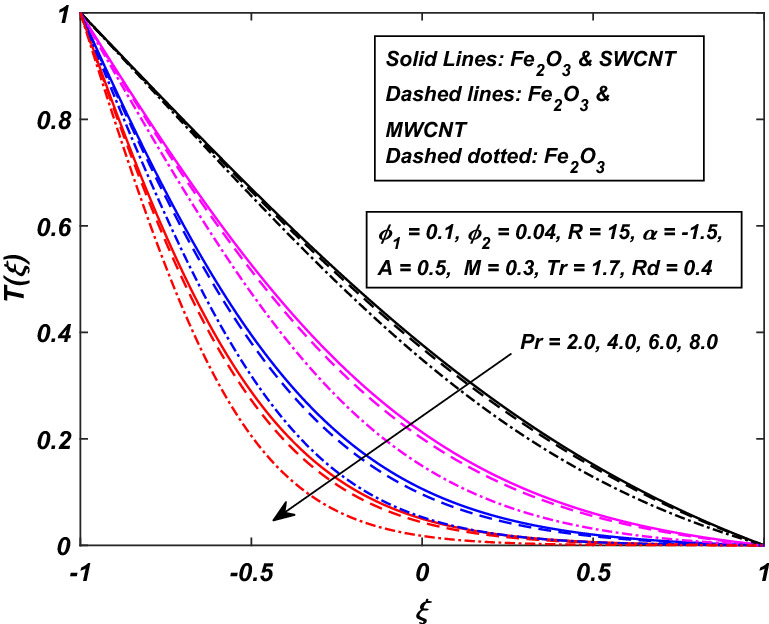
Influence of *Pr* on *T*(*ξ*).

The impact of temperature ratio parameter *Tr* (it is the ratio between the temperature at the wall and the reference temperature) is displayed in Fig. 14 for the variation in temperature distribution against the injection/squeezing channel. For the higher values of *Tr*, the temperature difference between the walls of the channel is high. A thinner thermal boundary layer is being seen as we push towards the upper wall. A considerable difference between the hybrid-nanofluid and Ferro-nanofluid is observed, as the hybrid shows a high temperature rise than the Ferro-nanofluid. The ratio between the momentum diffusivity to thermal diffusivity is famous as Prandtl number *Pr*. The effect of Prandtl number *Pr* on temperature profile has been displayed in Fig. 15. By raising the Prandtl number is actually the falling of thermal diffusivity of the fluid which ultimately reduces the temperature of the fluid. Reduction in temperature is more conspicuous in Ferro-nanofluid fluid.

The rate of local heat transfer is measured numerically for the different parameters. Tables are arranged for both hybrid-nanofluid (*Fe*_2_*O*_3_, *SWCNTs*, *MWCNTs*–*H*_2_*O*) and Ferro-nanofluid (*Fe*_2_*O*_3_–*H*_2_*O*). In Table 3, for the variation of Prandtl number, temperature ratio parameter, thermal radiation parameter, and porosity parameter, the numerical values are arranged against the local Nusselt number. Porosity parameter $A\,$ and Prandtl number *Pr* are the main factors that enhance the rate of heat transfer flow in the dilation channel when fluid is injected through the channel’s wall. This increment is much more dominant at the lower wall when compared with the upper wall. It is also clear from the table that Ferro-nanofluid has higher heat transfer rate than the hybrid-nanofluid. The same table represents the opposite influence of temperature ratio parameter *Tr* and thermal radiation parameter *Rd* against Nusselt number. Heat transfer rate on the channel wall decreases for the increasing values of *Tr* and *Rd*. Again this decrement is eminent at the lower wall. However, the rate of heat transfer is now reduced quickly for the hybrid-nanofluid when compared with Ferro-nanofluid. From the table, it is also noted that there is average 4.82% more rate of heat transfer is *Fe*_2_*O*_3_–*MWCNT* than the *Fe*_2_*O*_3_–*SWCNT*. Further, *Fe*_2_*O*_3_–*MWCNT*has 8.07% more heat transfer rate than the simple nanofluid.

**Table 3:** Numerical values of Nusselt number for various parameters, whereas *φ*_1_ = 0*–*05,*φ*_2_ = 0*–*04, *R* = 15, *a* = 0–5, *M* = 0–3.

*Pr*	*Tr*	*Rd*	*A*	*Fe*_2_*O*_3_–*SWCNT*		*Fe*_2_*O*_3_–*MWCNT*		*Fe*_2_*O*_3_–*H*_2_*O*	
				*Nu*_*lower*_	*Nu*_*upper*_	*Nu*_*lower*_	*Nu*_*upper*_	*Nu*_*lower*_	*Nu*_*upper*_
6.2	1	0.3	0.8	2.623501	0.044842	2.769287	0.036053	3.134645	0.017455
4				1.84859	0.133363	1.935411	0.117497	2.148572	0.078203
5				2.193489	0.082345	2.307227	0.069662	2.590674	0.040251
6				2.550931	0.04972	2.691424	0.04032	3.043361	0.020112
	1			2.714302	0.047109	2.85951	0.037961	3.146984	0.018976
	1			2.809421	0.049715	2.953687	0.040159	3.160771	0.020787
	2			2.907255	0.052712	3.050204	0.042694	3.176085	0.022949
		0.4		2.745532	0.057039	2.890202	0.046695	3.163819	0.02662
		0.5		2.85711	0.071039	3.000283	0.059086	3.194297	0.038379
		0.6		2.960355	0.086865	3.101779	0.073267	3.226246	0.052866
			0.5	2.066351	0.059296	2.16083	0.049048	2.391116	0.026111
			0.6	2.246722	0.053976	2.357563	0.044227	2.630923	0.02281
			0.7	2.43249	0.04918	2.560459	0.039917	2.878922	0.019946

In Table 4, we arranged the numerical values of local Nusselt number for volume fraction of nanoparticles $(\varphi_{1} ,\,\varphi_{2} ),$ local Reynolds number (suction/injection parameter) $R\,$ and channel’s wall deformation $\alpha .$ It is observed that by increasing the amount of ferricoxide nanoparticles in the base fluid, and if the injection rate is enhanced, the rate of heat transfer is significantly boosted. The lower wall exhibits more heat transfer when compared with the upper wall. Ferro-nanofluid has the least Nusselt number while MWCNTs have the most. On the other hand, the volume fraction of CNTs and the channel’s deformation rate are responsible for lowering the rate of heat transfer at the walls of the channel. Similar behavior is noted for the upper and lower walls. Decrement in the Nusselt number is more efficient in the case of hybrid-nanofluid. In Table 5, the numerical values are arranged to determine the skin friction coefficient on the lower and upper walls of the channel for the nanoparticles volume fractions $\varphi_{1} ,$ magnetic parameter $M,$ Reynolds number $R\,$ and channel’s wall deformation $\alpha .$ It is observed that nanoparticles volume fraction and Reynolds number have a direct relation with the skin friction whereas it is inversely related to magnetic parameter and wall deformation coefficient. It is further noted that skin friction increases more rapidly in the case of *SWCNT*–*Fe*_2_*O*_3_-water-based nanofluid when compared with *MWCNT*–*Fe*_2_*O*_3_ hybrid nanofluid.

**Table 4:** Numerical values of Nusselt number for various parameters, whereas *Pr* = 6–2, *Tr* = 1–7, *Rd* = 0–3, *A* = 0–8.

*φ*_1_	*φ*_2_	*R*	*α*	*Fe*_2_*O*_3_–*SWCNT*		*Fe*_2_*O*_3_–*MWCNT*		*Fe*_2_*O*_3_–*H*_2_*O*	
				*Nu*_*lower*_	*Nu*_*upper*_	*Nu*_*lower*_	*Nu*_*upper*_	*Nu*_*lower*_	*Nu*_*upper*_
0.1	0.04	15	0.5	2.623501	0.044842	2.769287	0.036053	3.134645	0.017455
0.1				2.985461	0.029317	3.144821	0.023107	3.708826	0.008024
0.1				3.350027	0.019319	3.522456	0.014942	4.285125	0.003736
0.1				3.71589	0.012857	3.901049	0.009768	4.861984	0.001771
	0.05			2.502922	0.049695	2.666787	0.038849	3.098331	0.016334
	0.06			2.393887	0.05427	2.571603	0.041487	3.062225	0.015309
	0.07			2.294553	0.05857	2.482773	0.043981	3.026315	0.01437
		10		1.876024	0.124614	1.964547	0.109201	2.181858	0.071339
		15		2.623501	0.044842	2.769287	0.036053	3.134645	0.017455
		20		3.411643	0.014903	3.613273	0.010942	4.119244	0.00387
			0.7	2.603902	0.044423	2.74759	0.0357	3.107179	0.017257
			0.9	2.584415	0.044006	2.72602	0.035348	3.079886	0.01706
			1.1	2.56504	0.043592	2.704578	0.034999	3.052766	0.016865

**Table 5:** Numerical values of Nusselt number for various parameters, whereas *Pr* = 6–2, *Tr* = 1–7, *Rd* = 0–3, *A* = 0–8.

*φ*_1_	*M*	*R*	*α*	*Fe*_2_*O*_3_–*SWCNT*		*Fe*_2_*O*_3_–*MWCNT*		*Fe*_2_*O*_3_–*H*_2_*O*	
				*C*_*flower*_	*C*_*fupper*_	*C*_*flower*_	*C*_*fupper*_	*C*_*flower*_	*C*_*fupper*_
0.1	0.3	15	0.5	2.146041	− 0.11815	2.080629	− 0.11912	1.998022	− 0.11715
0.1				2.172904	− 0.11896	2.108687	− 0.11991	2.028288	− 0.11788
0.1				2.194436	− 0.11964	2.131503	− 0.12056	2.053399	− 0.11848
0.1				2.210723	− 0.12019	2.149154	− 0.12108	2.073417	− 0.11895
	0.5			2.062765	− 0.13202	1.999258	− 0.13345	1.928057	− 0.12972
	0.6			2.026771	− 0.13906	1.964293	− 0.14071	1.897441	− 0.13608
	0.7			1.994158	− 0.14614	1.93274	− 0.14802	1.869464	− 0.14248
		10		1.494505	− 0.11948	1.451504	− 0.12059	1.395284	− 0.11896
		12		1.755221	− 0.11878	1.703257	− 0.11982	1.636457	− 0.11802
		14		2.015795	− 0.11832	1.954867	− 0.11931	1.877523	− 0.11739
			0.7	2.136697	− 0.11551	2.071516	− 0.11648	1.989586	− 0.11454
			0.9	2.127332	− 0.11293	2.062385	− 0.11389	1.981137	− 0.11198
			1.1	2.117944	− 0.11039	2.053235	− 0.11135	1.972672	− 0.10948

## Concluding remarks

The leading target is to contemplate the heat and flow characteristics of hybrid-nanofluid (*CNTs*–*Fe*_2_*O*_3_) with water *H*_2_*O* as carrier fluid through the rectangular asymmetric permeable horizontal parallel channel with an external applied magnetic field. The thermal radiation impact is also investigated. By varying the different physical parameters, flow and heat transfer features have been demonstrated with the help of graphs. The local rate of heat transfer is numerically calculated and arranged in the form of tables. A good comparative analysis is done for simple and hybrid nanofluid. The main features are as follows:

- Higher magnetic field shows in increment in flow speed between the expanding walls of the channel along with the injection of fluid. The fluid even gains more speed when we use Ferro-nanofluid instead of hybrid-nanofluid.
- For the squeezing walls of the channels, the temperature of the fluid rises when the thermal radiation parameter is enhanced. This enhancement is even more prominent in the case of hybrid-nanofluid (*CNTs*–*Fe*_2_*O*_3_, *H*_2_*O*).
- The influence of injection and suction is quite opposite for the temperature distribution. When more fluid is injected in the channel's wall, the temperature profile settled down a while in case of suction, it increases.
- In most of the cases, while computing the temperature distribution, SWCNTs are more dominant then MWCNTs.
- Local heat flux diminishes for the elevated thermal radiation parameter. It is higher for the Ferro-nanofluid and has lower values for the hybrid-nanofluid.
- Rate of local heat transfer at the lower wall is more prominent than at the upper wall.
- Increasing the nanoparticles volume fraction of *Fe*_2_*O*_3_ can increase the rate of heat transfer on the walls, but an opposite phenomenon is recorded for the single and multiwall CNTs.

## Data Availability

The data that support the findings of this study are available from the corresponding author.

## List of symbols

$$\tilde{p}$$: Pressure
$\tilde{v}$: Vertical component of velocity
$\tilde{u}$: Horizontal component of velocity
$$\overset{\lower0.5em\hbox{$\smash{\scriptscriptstyle\frown}$}}{y}$$: Vertical component of distance
$$\overset{\lower0.5em\hbox{$\smash{\scriptscriptstyle\frown}$}}{x}$$: Horizontal component of distance
$$\overset{\lower0.5em\hbox{$\smash{\scriptscriptstyle\frown}$}}{t}$$: Time
*C*_*p*_: Specific heat of fluid at constant pressure
*ρ*_*f*_: Density of carrier fluid
*ρ*_*hnf*_: Effective density of hybrid nanofluid
δ_*hnf*_: Thermal diffusivity of hybrid nanofluid
*µ*_*f*_: Viscosity of carrier fluid
*µ*_*hnf*_: Dynamic viscosity of hybrid nanofluid
*k*_*f*_: Thermal conductivity of carrier fluid
*k*_*hnf*_: Thermal conductivity of hybrid nanofluid
*σ*_*f*_: Electrical conductivity of carrier fluid
*σ*_*nhf*_: Electrical conductivity of hybrid nanofluid
*B*_*o*_: Magnetic constant
*α*: Wall's deformation rate
*A*_*l*_, *A*_*u*_: Permeability of lower and upper wall
*M*: Magnetic parameter
$$\mathop \sigma \limits^{*}$$: Boltzmann constant
*a*_*k*_: Mean absorbtion constant
*Pr*: Prandtl number
*Rd*: Radiation parameter
*R*: Reynolds number
$\tilde{T}$: Temperature of fluid
*T*_*r*_: Resistent temperature
*q*_*rad*_: Thermal radiation
